# Preventing Germinal Matrix Layer Rupture and Intraventricular Hemorrhage

**DOI:** 10.3389/fped.2013.00022

**Published:** 2013-09-05

**Authors:** Ronald W. Coen

**Affiliations:** ^1^Department of Pediatrics, St. Luke’s Regional Medical Center, Boise, ID, USA

**Keywords:** prematurity, brain, germinal matrix layer, intraventricular hemorrhage, platelets

## Abstract

The etiology of intraventricular hemorrhage (IVH) in extremely low birth weight preterm infants is multifactorial with circulatory instability and hemostasis being preeminent. This study sought to determine if the germinal matrix layer remained intact when platelets were above 200 × 10^9^/L, a near normal level, and fell below that when IVH occurred. This was a retrospective study of platelets and head ultrasounds (HUS) in infants 23–28 weeks gestation. Analyses were descriptive, one way analysis of variance, Pearson Chi-square tests, and *t*-tests. Platelet counts and HUS were linked in 114 infants during the first 3 days when 90% of IVHs occur. Mean platelet levels were >200 × 10^9^/L in 68% of infant 23–24 weeks gestation and 78% of those 25–26 weeks when there were no IVHs. These findings, if confirmed, suggest that improving hemostasis in high risk preterm infants by keeping platelet levels >200 × 10^9^/L may maintain the integrity of the germinal matrix layer and prevent IVHs.

## Introduction

**Figure 1:**
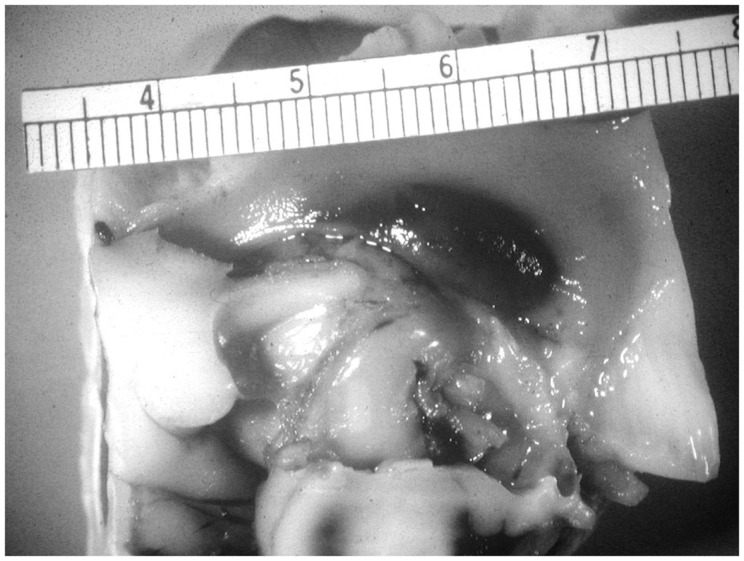
**Unruptured germinal matrix hematoma**. Photograph courtesy of Kevin Bove, and A. James Mc Adams (deceased), Department of Pathology, Cincinnati Children’s Medical Center.

The etiology of intraventricular hemorrhage (IVH) is multifactorial with prematurity, circulatory instability, and hemostasis being preeminent (1, 2). The presence of germinal matrix and its fragile vessels in the ganglion eminence makes the immature brain exceptionally vulnerable to hemorrhage, especially IVH (3). Efforts to prevent IVH have not been entirely successful in previous studies. Theoretically, IVH could be prevented by limiting germinal matrix layer hemorrhages (GMH) from enlarging and rupturing into the cerebral ventricles (Figure 1). Studies have suggested that extremely low birth weight (ELBW) infants may not be able to accomplish this because of their innately limited hemostatic capacity (4–6). One facet of their coagulation system that is not limited, however, is the concentration of circulating platelets. Accepted mean and median platelet levels in ELBW infants at birth are greater than 200 × 10^9^/L (7–11).

The role of platelets in the etiology of GMH and IVH has been questioned. Do lower platelets cause GMH-IVH or vice versa? In the absence of specific clinical conditions known to lower platelets, we postulated that platelet concentrations decrease as platelets are consumed when GMH rupture into the cerebral ventricles. This project was undertaken to study whether platelet concentrations remaining at or above 200 × 10^9^/L during the first 3 days following birth differentiate isolated germinal matrix layer hemorrhages from those that enlarge and rupture into the cerebral ventricles. If platelet levels at or greater than 200 × 10^9^/L distinguishes the infant without an IVH from one with, theoretically IVH could be prevented in high risk infants by keeping platelet concentrations higher than is currently practiced.

Waiting for platelets to fall to levels defining neonatal thrombocytopenia (<150 × 10^9^/L) before instituting platelet transfusions, as currently practiced, has not proven beneficial in preventing IVH in ELBW infants.

## Study Design and Methods

A retrospective review of the electronic medical records (NeoData^R^) of all preterm infants with gestational ages between 23 and 28 weeks was approved by our hospital’s Institutional Review Board. The records of 241 infants admitted to our Newborn Intensive Care Unit from January 1, 2006 through December 31, 2009, were reviewed. Parental consent was not required.

Platelet counts were performed on a Beckman Coulter Counter LH 750 or LH 500. The source of blood for the platelet counts could not be determined. Head ultrasounds (HUS) were performed on an Acuson Sequoia 512 ultrasound machine.

In order to achieve the study goal of determining whether higher platelet concentrations differentiated ELBW infants with no IVH from those with an IVH, we separated the five grades of IVH into groups defining events confined to the germinal matrix from those involving the cerebral ventricles. GMH and IVH were graded using the system of Papile et al. (12) HUS with no GMH were graded 0. The head ultrasound findings were separated into three groups according to whether the germinal matrix was not ruptured (grades 0–I) or had ruptured into an IVH (grades II–III) or into the parenchyma (grade IV). For the analysis the three groups of head ultrasound findings were analyzed according to platelet counts above and below 200 × 10^9^/L.

Stata version 10 was used for the analyses, which consisted of descriptive statistics, one way analysis of variance (ANOVA), Pearson Chi-square tests, independent samples *t*-tests, and paired *t*-tests (13). A *p*-value <0.05 was judged to be significant.

The investigator was not involved in the day to day management of any of the study infants.

## Results

We focused on clinical data available during the first 3 days since 90% of IVH occur during that time (1). There were no infants with alloimmune thrombocytopenia or platelet trapping tumors. Vitamin K administration is routine in our nurseries, but it was documented in only 80% of the medical records. The presence of central catheters was not documented. All but one infant received Ampicillin, which is reported to affect bleeding times (14).

Two hundred and forty one infants met the initial criteria for admission to the study. Twenty-two infants were excluded because they were transferred to the NICU after day 3, or they died within the first 2 days, or had only one platelet count in the first 3 days. Sixty-nine percent of the infants were Caucasian, 24% Hispanics, 1% blacks, and 7% a mix of Asian and other ethnicities. Forty additional infants were not included in the analysis because their mothers had pregnancy-associated hypertension (PIH), which is known to lower platelet numbers.

A definitive IVH grade was established by day 3 in 125 (70%) of the remaining 179 infants. For the final analyses we linked platelet levels that were performed on the same day or on the prior day, if no same day measurement was recorded, with the definitive HUS diagnosis. One hundred and fourteen (91%) of the 125 infants met these criteria. None received a platelet transfusion. The clinical characteristics of the 114 infants are shown in Table 1.

**Table 1:** **Clinical information for 114 infants between 23 and 28 weeks gestation**.

Sample characteristics	Platelets ≤200, *n* = 50	Platelets >200, *n* = 64	*P*-value
Birth weight (g)	845.4 (197)	875.7 (248)	0.48
Gestational age (weeks)	25.7 (1.5)	25.8 (1.4)	0.49
Male (%)	62.0	46.9	0.11
Apgar score <7 (%)	61.2	39.7	0.02
Small for gestation (%)	4.0	1.6	0.42
Multiple births (%)	32.0	26.6	0.53
Maternal hypertension (%)	6.0	3.1	0.46
Antenatal corticosteroids (%)	74.0	82.8	0.25
Cesarean section (%)	46.0	39.1	0.46
Severe RDS (%)	100	98.4	0.38
Pneumothorax (%)	4.0	3.1	0.80
Chorioamnionitis (%)	41.7	62.9	0.03
Any bruising (%)	48.0	34.4	0.14
Death (%)	29.8	15.9	0.08

*Values represent mean (SD) or percentages. RDS is respiratory distress syndrome*.

When the 114 infants were segmented by gestational age and IVH grades, 68.4% of the infants between 23 and 24 weeks gestation and 77.5% of the infants between 25 and 26 weeks with IVH grades 0–I had mean platelet levels >200 × 10^9^/L. Only 16.7 and 27.3% of the infants in the same age groups had platelets >200 × 10^9^/L with IVH grades II–III (*p* = 0.045 and 0.003, respectively). Platelet levels and IVH grades were not significantly associated in infants 27–28 weeks (*p* = 0.393).

**Figure 2:**
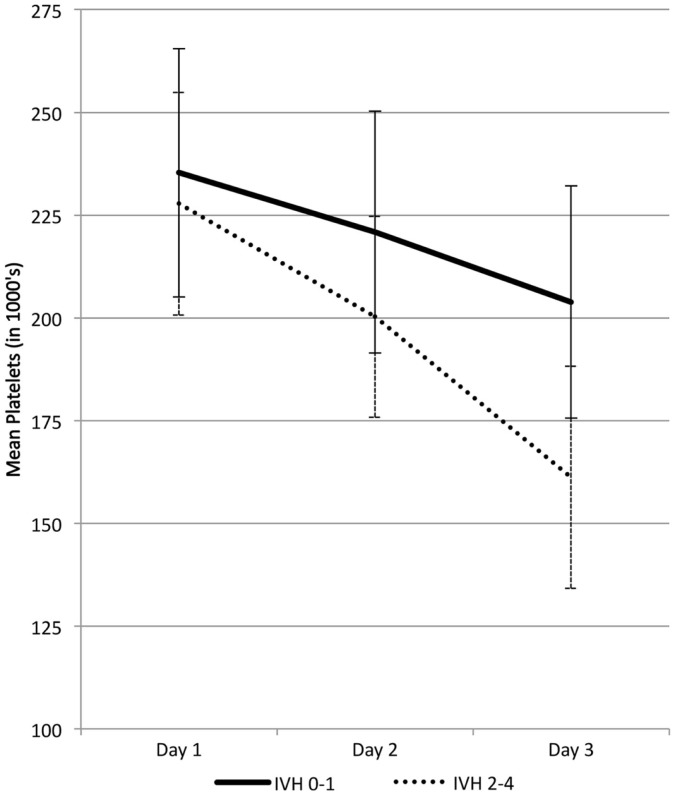
**Platelet levels (mean ± 2 SE) in 86 infants 26 weeks gestation or less with IVH grades 0–I and II–IV**.

Figure 2 shows mean platelet levels in infants 26 weeks gestation or less grouped according to their HUS diagnosis. Mean platelet levels were initially above or at 200 × 10^9^/L during the first 2 days in infants with all grades of IVH but by day 3 the mean level fell to between 150 and 175 × 10^9^/L when IVH grades II–IV were present (*p* < 0.001). Median platelet values were also significantly lower in infants with IVH grades II–IV compared to those with grade 0–I (*p* ≤ 0.001). Figure 2 also shows that the range of platelet values were never below 200 × 10^9^/L in infants with all grades of IVH on day 1, but by day 3 the lowest platelet values (<150 × 10^9^/L) were present in infants with IVH grades II–IV.

Coagulation studies were performed on only two infants. One infant with a tracheal culture positive for Group B streptococcus had a slightly prolonged prothrombin time (21 s), a normal partial thromboplastin time (108 s), and a normal fibrinogen level (299 mg/dL) on day 1. His platelets fell from 221 × 10^9^/L on day 1 to 189 × 10^9^/L when a grade II IVH increased to a grade IV on day 2 to 3. The second infant was not septic and had a grade I IVH on day 2 that progressed to a grade III by day 6. Coagulation studies on day 5 showed a normal prothrombin time (12 s), partial prothrombin time (44 s), and fibrinogen (261 mg/dL). His hematocrit fell from 40 to 27% and his platelets fell from 219 × 10^9^/L on day 1 to 103 × 10^9^/L on day 3.

Neonatal hypotension, defined as a mean blood pressure lower than that expected for gestational age, was associated with lower platelet levels and higher grades of IVH by day 3 (*p* < 0.01). Apgar scores <7 and chorioamnionitis also significantly associated with lower platelet counts and IVH (Table 1). Neonatal sepsis was not related to IVH.

## Discussion

In order to reduce or prevent IVH, special attention must focus on maintaining the integrity of the germinal matrix layer. Past studies of IVH have not separated germinal matrix events from other grades of IVH, as was done in this study. Most investigators use a level of 150 × 10^9^/L or less as the benchmark for linking platelets with IVH. The results of the current study show that on days 1 and 2 this level is low. By day 3 we observed continued lowering of the platelet concentration suggesting that intraventricular bleeding has occurred and platelets are being consumed. Conceivably, micro-hemorrhages in the germinal matrix layer could be prevented from progressing into IVH by transfusing platelets in high risk infants soon after birth and maintaining the level above 200 × 10^9^/L during the first 3 days.

In this study the germinal matrix layer of 68–78% of the infants 23–26 weeks gestation remained intact when mean platelet levels were at or above 200 × 10^9/^L during the first 3 days. By comparison infants with IVH grades II–III and IV had platelets below that level. This finding suggests that platelet levels at or above of 200 × 10^9^/L define the dynamic hemorrhagic events more critically in the immature brain than levels below 150 × 10^9^/L, the current definition of neonatal thrombocytopenia. Further, waiting for platelet levels to fall below 50 × 10^9^/L to meet the current guideline for platelet transfusions in preterm infants may be too late to protect the germinal matrix layer and prevent IVH (15–18).

Platelet changes are reported to occur on day 2 in infants with IVH (19, 20). We also found that platelets fell more rapidly on days 2–3 when an IVH grade II or higher was present. These three studies stress the importance of following platelets, hematocrit, and coagulation values closely during the first 3 days in ELBW infants that are at high risk for developing IVH.

The present study did not answer whether lower platelet levels precede or are the consequence of IVH. Exposure to Ampicillin and other conditions that have been associated with bleeding and the etiology of IVH were addressed. Overall, in the absence of other identified platelet consuming diseases, it seems reasonable to suggest that in our study platelets were consumed more rapidly once bleeding extended into the cerebral ventricles. Others have reported that IVH occurs in association with a consumptive coagulopathy and increased platelet destruction (21, 22).

Extremely low birth weight infants are at risk for hemorrhaging because their platelets are hypoactive and bleeding times are long, especially on day 1 (5, 6, 23, 24). Bleeding times, although difficult to measure, reportedly improve with transfusions of adult platelets (25). In another retrospective investigation platelet mass was studied to determine its relationship to IVH, since decreased platelet mass is known to increase bleeding times (26). When platelet mass, the product of mean platelet volume times platelet concentration, fell to <10th percentile (1144 fl nL^−1^) on day 2, which was day 3 in our study, severe grades of IVH and increased mortality were present. Unlike our study, the data in the referenced study were not linked to those at highest risk for IVH, specifically infants 26 weeks or less. Including larger and more mature preterm infants in studies of IVH dilutes the importance of findings related specifically to the highly vulnerable extremely preterm infant. The role of platelet mass in differentiating static GMH from those progressing to IVH and the use of platelet mass as an indicator for platelet transfusion requires further study, especially in infants <26 weeks gestation.

Andrew et al. first reported that thrombocytopenia, defined as a platelet level <150 × 10^9^/L, increased the risk of IVH (27). They then conducted the only prospective randomized controlled trial of platelet transfusions in an attempt to prevent IVH (25). Transfusions of adult platelets were administered when platelet levels fell below 150 × 10^9^/L. Infants were transfused at a mean age of 1.92 days, about the same time that platelet levels were falling in our study and in those of von Lindern et al. and Piotrowski et al. (19, 20). Following transfusions, platelet levels rose and remained above or near 200 × 10^9^/L. After re-grouping the Andrew’s data, it appears that the percent of transfused infants with IVH grades 0–I remained about the same on both initial and final HUS, so they may have prevented some GMHs from progressing to IVH. Bleeding times shortened in transfused infants but little was achieved in reducing the number of infants with grade II and III hemorrhages pre-and post transfusion (25). We believe that platelet transfusions in their study were administered at a time when germinal matrix rupture and intraventricular bleeding were underway. By then, it was too late to prevent IVH.

In a recent publication, von Lindern and her colleagues showed that the outcome of severe grades of IVH (grade III or IV) were the same in preterm infants <32 weeks gestation whether a restrictive platelet transfusion protocol or a more liberal protocol was used (28). Neither protocol required platelet transfusions at levels of 200 × 10^9^/L. It appears that preventing IVH can only be achieved by limiting GMHs from enlarging and rupturing. Waiting for platelets to reach a lower level before considering platelet transfusions may not be the method for preventing IVH.

Like most retrospective studies, this one also had major drawbacks. First and foremost was the small study population which was due mainly to HUS and platelet determinations not being performed in all cases on the same day and within hours of each other. Some records did not provide information about Vitamin K administration even though it is a stated policy in our unit. In addition, the use of central catheters was not always clearly stated. Nevertheless, the findings provide a starting point for a prospective study in which HUS and platelet counts are temporally related during the first 3 days. If the findings of such a study confirm those in this study, a randomized prospectively designed protocol should be considered. Knowing that increased mortality and morbidity have been reported with four or more platelet transfusions, the study design should employ early and a limited number of platelet transfusions.

Finally, current guidelines recommend platelet transfusions for sick newborns when platelet levels are <50 × 10^9^/L and there is active bleeding. These guidelines may not apply to the management of ELBW infants at high risk for IVH for several reasons. First, there are no current scientifically based studies to support the transfusion recommendations (16–18). Second, transfusing preterm infants with adult platelets improves their bleeding times, so why not try to stop a GMH before it segues into an IVH? Third, one of the guidelines for transfusing platelets is evidence of active bleeding. GMH-IVH can only be detected by ultrasound scans and even then scans do not show active bleeding. The results of the present study suggest that keeping platelets above 200 × 10^9^/L rather than waiting for them to fall below 150 × 10^9^/L may provide a clinical opportunity to prevent IVH.

## Conclusion

This preliminary study showed that platelet concentrations at or above 200 × 10^9^/L during the first 3 days defines the threshold for differentiating events in the germinal matrix layer and IVH in the immature brain. If the results are confirmed, future trials may be designed to show whether IVH can be prevented by improving the hemostatic capacity of the ELBW infant with early and a limited number of platelet transfusions, administered either prophylactically or as active treatment.

## Conflict of Interest Statement

The author declares that the research was conducted in the absence of any commercial or financial relationships that could be construed as a potential conflict of interest.
